# Polychlorinated Biphenyl Profile in Polyhydroxy-alkanoates Synthetized from Urban Organic Wastes

**DOI:** 10.3390/polym12030659

**Published:** 2020-03-14

**Authors:** Carmela Riccardi, Francesca Buiarelli, Federica Castellani, Patrizia Di Filippo, Laura Lorini, Mauro Majone, Mariana Matos, Donatella Pomata, Giulia Simonetti, Bruno Sommer Ferreira, Francesco Valentino

**Affiliations:** 1 INAIL–DIT, Via Roberto Ferruzzi 38, 00143 Rome, Italy; p.difilippo@inail.it (P.D.F.); d.pomata@inail.it (D.P.); 2 Department of Chemistry, University of Rome “La Sapienza”, P.le A. Moro 5, 00185 Rome, Italy; francesca.buiarelli@uniroma1.it (F.B.); federica.castellani@uniroma1.it (F.C.); laura.lorini@uniroma1.it (L.L.); mauro.majone@uniroma1.it (M.M.); giulia.simonetti@uniroma1.it (G.S.); francesco.valentino@uniroma1.it (F.V.); 3 Chemistry Department, Faculdade de Ciências e Tecnologia-Universidade Nova de Lisboa, 2829-516 Caparica, Portugal; m.matos@campus.fct.unl.pt; 4 Biotrend SA, Biocant Park, Nucleo 04 Lote 2, 3060-197 Cantanhede, Portugal; bsferreira@biotrend.pt

**Keywords:** polyhydroxyalkanoates, municipal organic wastes, polychlorinated biphenyls

## Abstract

The microbial synthesis of polyhydroxyalkanoates (PHA) from organic wastes is a valuable process to valorize available renewable resources, such as food wastes and biological sludge. Bioplastics find many applications in various sectors, from medical field to food industry. However, persistent organic pollutants could be transferred from wastes to the final product. The present paper demonstrates that the use of municipal wastes in PHA production is safe for the environment and human health and provides a polychlorinated biphenyl (PCB) profile in both commercial and waste-based PHA samples. PCB analysis in several PHA samples showed very low concentrations of the target analytes. Commercial PHA samples showed a similar PCB level with respect to PHA samples from municipal waste/sludge and higher than PHA samples from fruit waste. For all analyzed PCBs, detected concentrations were consistently lower than the ones reported in regulatory framework or guidelines.

## 1. Introduction

Polychlorinated biphenyls (PCBs) are organic pollutants widely distributed in THE environment. They are highly toxic compounds that can cause adverse effects on humans and animals, such as cancer, nervous system damage, reproductive disorders and disruption of the immune system [1]. In 2016, the International Agency for Research on Cancer (IARC) updated the classification of PCBs to Group 1 (carcinogenic to humans) from the previous Group 2A (probably carcinogenic to humans) [2].

Due to their high chemical and biological stability and lipophilicity, PCBs can be considered persistent organic pollutants (POPs) that bioaccumulate in the environment and along the food chain [3]. They are included in the Stockholm Convention that has banned the use and the intentional production of these compounds [4,5].

In the past, PCBs were used extensively as hydraulic fluids, flame retardants, plasticizers, etc. [6] and, to date, they can still be released in the environment following the dismantling of old devices, the disposal of manufactured products and the recycling of electrical and electronic equipment [7,8,9]. Moreover, PCBs can be produced unintentionally by thermal processes and incomplete chemical reactions [6,10,11]. Consequently, they can be still detected in environmental media and their occurrence was found in soil, sediments, water, vegetation, municipal wastes, compost, etc. [11,12,13,14].

In particular, Hellström et al. [15] investigated PCB occurrence in the biodegradable fraction of source separated household wastes, compost, and anaerobic digestate. They proved that some congeners degrade at a similar rate as the waste is mineralized, and similar concentrations were found in both waste and derived products; on the other hand, other PCBs were detected in feedstock and compost, but not in digestates. Barret et al. [16] identified the sorption of PCBs on organic matter as main mechanism justifying the occurrence of these compounds in biological sludge produced in municipal wastewater treatment plants (WWTPs). Moreover, the authors demonstrated that sorption phenomena redistributed PCB fluxes throughout physical separation units and, as consequence, in the solid and liquid effluents.

In December 2015 the European Commission launched the Circular Economy Package [17], which aims at supporting European businesses and consumers to make the transition to a stronger and more circular economy where resources are used in a more sustainable way. The CE Package includes a common EU target for recycling 65% of municipal waste by 2030. In this perspective, among “hot spots” of using waste as raw material, possible content of POPs that can migrate from waste into end-products is a matter of concern. The possible environmental spreading of pollutants when reusing or recovering waste is cause of scientific debate and major controversy still at regulatory level and still waiting to be completely defined. As an example, the agronomic reuse of compost, wastewater sludge and/or anaerobic digestate as soil improvers is presently the main way to recover nutrients from waste streams but also subjected to limitations [18]. These concerns hold to a greater reason when dealing with bio-waste coming from sources that can be neither easily segregated nor completely controlled, such as waste and sludge of urban origin. 

In this frame, novel approaches and technologies are being developed to obtain a more diverse portfolio of bio-based products other than soil improvers and biogas. This calls for fully reconsidering the matter of contaminant migration in waste “recycling” by taking into account that different technologies and novel products can completely change the scenario. As an example, the use of organic wastes to produce biopolymer such as polyhydroxyalkanoates (PHAs) by microbial fermentation processes is a widely studied approach [19]; however, PCBs could be present in bioreactor feedstock and be transferred to the final product. Since PHA-based bioplastics can be used in biomedical applications, to produce multilayer food packaging, durable goods etc. [20,21,22], product safety has to be guaranteed. Therefore, a reliable determination of PCB content in PHA is required. 

Many analytical methods have been developed for analysis of PCBs in air, water, soil, sediments, wastes and biological tissues and fluids [6]. These methods usually involve a first extraction step that can be a Soxhlet extraction, an accelerated solvent extraction (ASE), an ultrasonic solvent extraction (US) or a microwave-assisted extraction (MAE); a subsequent clean-up of extracts, mostly carried out by alumina, silica gel or Florisil chromatography columns; and lastly gas chromatography–mass spectrometry (GC–MS) analysis [6]. Lately, analytical procedures that combine extraction and clean-up steps have been developed. These methods involve reduced organic solvent consumption as well as reduced extraction and analysis time, and less sample handling while providing performance comparable to conventional sample preparation methods [23,24,25].

To the best of authors’ knowledge, there are no reports on PCB determination in waste-derived PHA samples. Therefore, the aim of the present paper is to provide a PCB profile in PHA samples obtained from different organic wastes (food waste and/or biological sludge) and to evaluate if PCB detected concentrations meet regulatory standards and guidelines for the environment and human health in order to offer a sound management and valorization of wastes. A one-step procedure that combines extraction and clean-up was applied. Gas chromatography–negative chemical ionization–mass spectrometry (GC/NCI–MS) was used for PCB analysis. Since the procedure was developed for environmental samples [9], it was necessary to assess method performance with polymer matrix and potential interferences due to matrix components were estimated. Lastly, samples of PHA produced in two bioreactors fed with organic fraction of municipal solid waste (OFMSW)/biological sludge and wastes from fruit processing industry were analyzed in order to verify if use of municipal wastes in PHA production is safe for the environment and human health. 

## 2. Experimental

### 2.1. PHA Production and Extraction

PHA samples were obtained from two pilot platforms located in Treviso (northeast Italy) and Lisbon (Portugal). The Treviso pilot plant (TV) feedstock was a mixture of liquid slurry from squeezing the organic fraction of municipal solid waste (OFMSW) and biological sludge from urban wastewater treatment. The detailed process is given in Valentino et al. [26]. Briefly, the main process steps were a first anaerobic fermentation reactor for PHA-precursor (volatile fatty acid, VFA) production, a second aerobic reactor (sequencing batch reactor, SBR) for biomass cultivation, and a third fed-batch aerobic reactor for PHA accumulation within cellular wall. At the end of this phase, PHA-rich biomass was collected, stabilized, stored, and finally addressed to the extraction/purification steps. The total mass yield was equal to 7.6% gPHA/g volatile solids (VS) [27]. 

After PHA accumulation and before extraction, PHA-rich biomass samples were stabilized by either thermal drying or acidification. In the first case, the PHA-rich biomass was left to settle under gravity and then the thickened slurry was centrifuged (15 min at 4500 rpm, Heraeus Megafuge 40, Thermo Fisher Scientific, Waltham, MA USA). The wet pellet was thermally pre-treated at 145°C for 15 min, and then dried overnight in the same oven at 60 °C. In the second, the slurry containing PHA-rich biomass was directly acidified with H_2_SO_4_ down to pH 2.0 and left to settle under gravity overnight. The thickened slurry was then centrifuged (5 min at 4500 rpm, Heraeus Megafuge 40) and the wet pellet stored in the fridge (4 °C).

PHA production process developed in Lisbon pilot plant (LB) was similar to the TV one. The main difference was the feedstock, which, in LB, was fruit processing industry waste. The pilot units consisted in three reactors: an upflow anaerobic sludge bioreactor (UASB), a sequential batch reactor (SBR) and a stirring tank reactor operating in fed-batch mode. Fermented fruit waste was the feedstock for both biomass cultivation and PHA accumulation step. In the LB case, the PHA-reach was stabilized through acidification only.

PHA extraction from biomass was performed by three different methods. Extraction solvents used (chloroform, hypochlorite and chlorine free aqueous phase inorganic reagents) represented the main difference. Both extraction methods with chloroform and hypochlorite were applied only to thermally dried PHA-rich biomass produced in TV. For the first one, a 2–5 g sample was put into a glass fiber extraction thimble placed inside of a 50 mL borosilicate glass Soxhlet extractor. The Soxhlet extractor was equipped with a 100 mL borosilicate glass flask, filled with 70 mL of chloroform, a bubble condenser and a boiler, in order to keep the solvent at boiling temperature for 24 h. The PHA-enriched solvent was cooled down at room temperature and then recovered into a crystallizer to obtain a polymer film by solvent evaporation. In order to minimize PCB abatement through the different downstream processing steps, PHA precipitation in a non-solvent was not carried out.

For extraction with hypochlorite, dried PHA-rich biomass was suspended in a NaClO solution (1.5% active Cl_2_). The suspension was kept under magnetic stirring overnight, then centrifuged at 8500 rpm for 20 min and washed 3 times with distilled water. Wet pellet was finally transferred into a crystallizer and oven dried at 70 °C. 

Extraction method with inorganic reagent was carried out on acid-stabilized wet biomass coming from several batch tests performed in both TV and LB. At the end of the accumulation test, PHA-rich biomass was acidified, centrifuged and stored at 4 °C. Extraction with a mixture of chlorine-free aqueous phase inorganic reagents was applied following a reserved protocol optimized by Biotrend SA (Cantanhede, Portugal) that is a research-based company specialized in the development of bioprocesses. At the end of the extraction, the polymer was dried obtaining a white powder.

### 2.2. PHA Characterization

Monomer content of PHA samples was determined in agreement with Braunegg et al. [28]. The polymer was extracted, hydrolyzed, and esterified to 3-hydroxyacyl methyl esters and quantified by GC method. The abundance of 3-hydroxybutyric (3HB) and 3-hydroxyvaleric (3HV) monomers was quantified by P(3HB-co-3HV) standard polymer at 5 wt.% HV content (Sigma-Aldrich, Milan, Italy).

The molecular weight was determined through viscosimetric analysis. The equipment for the measure of the gravimetric flow time of each solution was composed by a AVS 350 viscosimeter (SCHOTT, Letchworth, Great Britain) equipped with an AVS/SHT sensor, a CD15 thermostatic bath, working at 30 °C (LAUDA, Frankfurt, Germany) and a SCHOTT GERÄTE Ubbelohde capillary viscosimeter (ID = 0.46 mm). For each sample, 15 mL of solution 0.5% w/v were transferred in the Ubbelohde capillary viscosimeter and the flow time was measured through the optical sensor. Then, at least 4 dilutions were made directly in the viscosimeter by adding predetermined aliquots of solvent. From the flow time of each solution, the intrinsic viscosity [*η*] of polymer samples was determined. The viscometry average molecular weight ($M_{v})$ was calculated according to the Mark-Houwink equation:

(1) $$M_{v}\to \left[\eta \right]=K\cdot \;{M_{v}}^{\alpha }$$

where, K = 7.7 · 10^−5^ and α = 0.82 [29].

The thermal stability or degradation temperature (*T*_d_) of the samples was evaluated by thermogravimetric analysis (TGA) using a TG50 thermobalance (Mettler, Milan, Italy). Approximately 5 mg of dried samples were weighted on the balance. The analysis was conducted in nitrogen flow (20 mL/min) by heating the samples from 30 °C to 500 °C at 10 °C/min.

### 2.3. Sample Preparation

Extraction of analytes from PHA samples was performed by an ASE 200 accelerated solvent extractor (Dionex, Sunnyvale, CA, USA) equipped with 11 mL stainless-steel cells. In agreement with a previous study [9], extract clean-up was carried out simultaneously to the extraction (“in-cell clean-up”) filling the cell with Florisil as sorbent. 10 mg of sample were mixed with 500 mg of Florisil, spiked with 10 μL of PCB internal standard solution (^13^C marked PCB60 and ^13^C marked PCB153, 0.5 ppm) and extracted twice with ASE200. Operating conditions were: system pressure = 1500 psi; purge nitrogen pressure = 150 psi; purge nitrogen time = 300 s; oven temperature = 100 °C; oven heat-up = 5 min; static time = 5 min; flush volume = 60% of extraction cell volume; n-hexane 100%. The extraction of biopolymer samples was carried out in triplicate. The extracts were evaporated to dryness under a gentle nitrogen stream with an SE500S evaporator (Dionex). Operating conditions were: N_2_ temperature 35 °C, plate temperature 35 °C, stirrer 120 rpm, duty cycles 90%, pulse/min 70. The samples were re-dissolved with 50 µL of toluene and stored at −20 °C in amber vials until analytical determinations.

### 2.4. GC–MS Analysis

PCBs were analyzed using an Agilent Technologies 7890B gas chromatograph with a 5977B mass selective detector (Agilent Technologies, Palo Alto, CA, USA) operating in negative chemical ionization (NCI). GC separation was carried out on an HP5–MS (5% phenyl 95% dimethylpolysiloxane, 30 m × 0.25 mm i.d., 0.25 μm film thickness) fused silica capillary column (Agilent Technologies). One μL splitless injections were applied with an injector temperature of 280 °C. Oven temperature program was as follows: 100 °C increasing at 25 °C/min to 310 °C held for 8 min. The helium carrier gas was at a constant flow of 1 mL/min. Quadrupole, ion source and transfer line temperatures were set at 150, 230 and 300 °C, respectively. The reagent gas was methane at 40 mL/min. The MS was operated in selected ion monitoring (SIM) mode for quantitation of target compounds. All the MS parameters are reported in [9]. The PCB analyzed were: PCB28, PCB77, PCB81, PCB99, PCB101, PCB105, PCB110, PCB114, PCB126, PCB138, PCB146, PCB151, PCB156, PCB157, PCB167, PCB169, PCB170, PCB177, PCB180, PCB183, PCB187, PCB190. All the standards were obtained from Wellington Laboratories Inc. (Guelph, ON, Canada). The target compounds were selected for their toxicity and relative abundance in environmental samples as suggested by other authors, official methods and Stockholm Convention [4,30,31,32]. The analytes were identified on the basis of their mass spectra using the base peak and at least one qualifier ion depending on the compound and quantified by internal standard method and matrix-matched calibration curves. Quality control, consisting of blank measures and calibration verifications, was carried out routinely.

### 2.5. Calibration Curves

Both matrix-free and matrix-matched calibration curves were built to evaluate limit of detection (LOD) and limit of quantitation (LOQ), interday and intraday reproducibility, and matrix effects. Matrix-free calibration curves consisted of six standard solutions with increasing PCB concentrations in the range 6–600 mg·L^−1^ depending on the analyte. For the matrix-matched calibration curves, known quantities (30 mg) of three commercial PHA were grinded, mixed and homogenized to obtain a representative sample of the several biopolymer matrix. Then, six aliquots of 10 mg of PHA mixture were spiked with PCB multistandard solutions in the range 6–600 mg·L^−1^ depending on the analyte. The spiked polymer samples were subjected to the procedure described in par. 2.2 and calibration curves were built. PCB concentrations were below LOD except for PCB126, 138, 169, 180 and 187. For these compounds, linear extrapolation of the calibration curves to zero signal yielded their concentrations. Since the values were not negligible, these curves were translated to the origin in order to obtain the matrix-matched calibration curves need to quantify the analytes in PHA samples. For all the calibration curves, the solutions were prepared in triplicate and injected three times in GC/NCI–MS. Furthermore, quality control standards and blanks were analyzed on each day.

## 3. Results and Discussion

### 3.1. Preliminary Assessment of PHA Composition, $M_{v}$ and T_d_

The monomer composition (3-hydroxybutyrate and 3-hydroxyvalerate; 3HB–3HV wt.%), $M_{v}$ and *T*_d_ were quantified for each type of polymer. In both pilot scale platforms, a copolymer poly-3-hydroxybutyrate-co-3-hydroxyvalerate [P(3HB)-co-(3HV)] at different 3HB–3HV content (wt.%) was obtained. 

The PHA produced in TV and extracted with chloroform had a relative monomer content in the range 7.7–20.2 wt.% for 3HV and 79.8–92.3 wt.% for 3HB; $M_{v}$ and *T*_d_ were 110–170 kDa and 290–303 °C, respectively. PHA produced in TV and extracted with NaClO solution had similar composition and comparable properties to those samples extracted in chloroform: 7.0–19.7 wt.% for 3HV and 80.3–93.0 wt.% for 3HB; $M_{v}$ and *T*_d_ were equal to 85–180 kDa and 269–287 °C respectively. 

Regarding the polymer extracted with the Biotrend method, the PHA produced in TV had the following properties: 3HV 14.7–22.5 wt.% and 3HB 77.5–85.3 wt.%; $M_{v}$ 321–408 kDa; T_d_ 267–292 °C. The PHA produced in LB exhibited a narrow range of composition and properties: 3HV 7.4–7.7 wt.% and 3HB 92.3–92.6 wt.%; $M_{v}$ 248–256 kDa, T_d_ 270–272 °C. 

### 3.2. Method Performance

Since the analytical procedure was developed for environmental samples [9], it was necessary to assess method performance with the polymer matrix. Therefore, in the absence of certified reference materials of matrices similar to PHA polymer, an in-house reference material consisting of commercial polymer samples spiked with analytes was prepared as recommended by AOAC International and other authors [33,34,35]. 

Table 1 shows the LOD, LOQ and reproducibility of the method. LOD is the concentration at which signal to noise ratio (S/N) for the quantifier ion is >3, whereas LOQ is the concentration for which the S/N is >10. Determination of S/N was performed using serial dilutions of commercial polymer samples spiked with known concentrations of analytes and comparing measured signals with those of blank samples [36]. Both diluted spiked samples and blanks were processed according to analytical described procedure. Reproducibility is expressed as relative standard deviation (RSD) of six consecutive measurements of samples spiked with target analytes and processed with the matrix-specific methodology in the same day (intra-day) or in five different days (inter-day). 

Since any LOD and LOQ value was available for PCB analysis in PHA samples, the results were compared to those of analytical methods applied to environmental matrices, as suggested by other authors [24]. LOD and LOQ values are consistent with previous studies [25,37,38] and are much lower than law limits reported in regulatory framework or guidelines [39,40,41]. For PCB99, higher LOD and LOQ were measured likely due to method poor sensitivity for this compound. Nevertheless, they still allow to determine its concentration below the limits imposed by most legislation. Furthermore, PCB99 is one of the major congeners detected in wildlife and humans, therefore its content in environmental matrices is almost never negligible [42].

The intra-day and inter-day RSD for the compounds ranged from 1% to 12% and from 2% to 20%, respectively, indicating good reproducibility of method as values ≤20% can be considered acceptable for complex analytical methods. 

Table 2 shows recoveries for each analyte and matrix effects. As for recovery percentages, before the extraction PHA samples were fortified with the compounds and processed according to analytical procedure described in 2.2. All the PCB were spiked at 5 μg/kg except PCB99 that was added at 40 μg/kg and PCB28 at 15 μg/kg. The internal standards were added prior to GC–MS analysis. PCB concentrations were calculated with matrix-matched calibration curves and the recovery was determined by the following formula: *C*_exp_/*C*_n_ × 100, where *C*_exp_ is the experimental concentration and *C*_n_ is the nominal one.

Matrix effects (ME) were calculated as percentage between the slope of calibration curve obtained with matrix-matched standards (MMS) and the one obtained with solvent standards. 

The good recoveries demonstrate that no significant loss occurs at any stage of the analytical procedure and no further clean-up is necessary. In addition, the method allows low manipulation of the sample, reduced solvent consumption and lowered extraction and analysis time. Many authors [23,24,25,37,38] applied analogous extraction and purification methods on solid environmental matrices yielding comparable results, but this is the first time that this analytical procedure is carried out on biopolymer samples. 

Although GC–MS suffers less from ME than liquid chromatography tandem mass spectrometry, several authors report evidence and suggest methods to correct these effects [43,44,45,46]. Both interaction and accumulation of matrix components in the injector liner and in the GC column may lead to alterations of analyte signal [45]. Consequently, ME can result in either suppression or enhancement of the compound signal intensity. As suggested by other authors, percentages greater than 110% and lower than 75% were considered indicative of signal enhancement and suppression, respectively [25,47]. The severe signal suppression for PCB28, 77 and 110 strongly suggests the use of MMS to perform an accurate quantitation. Likewise, the signal enhancement for the other PCB requires to work with matrix-matched calibration curves in order to ensure reliable results. 

### 3.3. PCB Concentrations in PHA Samples

Table 3 and Table 4 show PCB concentrations in PHA samples of different origin. All the concentrations are expressed as mean value ± standard deviation.

Although PCBs were found in all the PHA samples, their concentration was very low and usually below 10 µg/kg. The only exception is for PCB99 in agreement with its relative abundance in wildlife and humans [42]. Its concentration was about 50 µg/kg in the samples coming from municipal waste/sludge and stored after thermal drying, regardless of the extraction method. Commercial PHA showed a similar PCB99 content, as well. Conversely, acid treatment for storage of polymer samples produced from both municipal waste/sludge and fruit waste leads to a concentration of PCB99 under the detection limit.

For the other PCBs, biopolymers from fruit waste were the least contaminated samples and only three out of 21 PCBs (PCB138, PCB169, and PCB(170 + 190)) showed concentrations above the detection limit. The PHA samples coming from municipal waste/sludge showed detectable concentrations of almost all PCBs, regardless of whether the polymer was extracted or not and the method of extraction. Once again, commercial PHA samples showed a similar PCB level with respect to PHA samples from municipal waste/sludge and higher than PHA samples from fruit waste.

In addition, waste-based PHA samples which underwent acid pretreatment for stabilization and then chlorine free aqueous-phase extraction had lower PCB levels than commercial PHA samples, and especially if coming from fruit waste.

Dioxin-like PCBs exhibited low concentrations in all the samples, ranging from 1 to 3.4 μg/kg. Furthermore they were undetectable in PHA samples from fruit waste with the exception of PCB169. 

For all analyzed PCBs, detected concentrations were consistently lower than the ones reported in regulatory framework or guidelines.

As an example, dealing with Recycling Plastics from Shredder Residue, EPA is adopting the “generic 50 ppm (i.e., 50 mg/kg) exclusion from the ban for the processing, distribution in commerce, and use of PCBs, based on the Agency’s determination that the use, processing, and distribution in commerce of products with less than 50 ppm PCB concentration will not generally present an unreasonable risk of injury to health or the environment” [41]. It is noteworthy that this threshold is orders of magnitude higher than PCBs found in waste-based PHA samples. 

The US Food and Drug Administration (FDA) also limits PCBs in paper food-packaging materials to 10 ppm (mg/kg) [39].

## 4. Conclusions 

In the context of the circular economy and in line with innovative waste management practices, the present paper provides a PCB profile in PHA samples obtained from food wastes and biological sludge. In all the PHA samples, PCB concentrations were consistently lower than the ones reported in the regulatory framework. Therefore, the use of such waste as raw material for PHA production process appears to be safe for the environment and human health. This paves the way for a possible market use of PHA synthetized from waste, with new opportunities for the management of wastes by their conversion into high-added value products.

## Figures and Tables

**Table 1:** LOD, LOQ and intra- and inter-day reproducibility of method.

Compound	LOD (μg·kg^−1^)	LOQ (μg·kg^−1^)	RSD Intra-Day	RSD Inter-Day
**PCB28**	3	11	1.8	3
**PCB77**	0.12	0.42	1.1	2
**PCB81**	0.14	0.47	6.9	15
**PCB99**	9.8	30	1.8	14
**PCB101**	0.09	0.3	8.1	11
**PCB105**	0.06	0.2	12	19
**PCB110**	0.17	0.56	11	20
**PCB114**	0.08	0.27	4.8	9
**PCB126**	0.12	0.41	2.8	10
**PCB138**	0.11	0.37	8.4	15
**PCB146**	0.10	0.35	3.6	17
**PCB151**	0.08	0.27	4.8	8
**PCB156**	0.13	0.42	2.2	5
**PCB157**	0.11	0.37	2	5
**PCB167**	0.12	0.40	0.9	6
**PCB169**	0.12	0.39	0.6	3
**PCB(170 + 190)**	0.46	1.55	0.8	18
**PCB177**	0.11	0.38	3.9	6
**PCB180**	0.10	0.34	1	8
**PCB183**	0.13	0.43	1.8	13
**PCB187**	0.09	0.31	5.7	14

**Table 2:** Recoveries and matrix effects (ME) of analytical procedure for each PCB.

Compound	Recovery (%)	ME (%)
**PCB28**	89	44
**PCB77**	95	10
**PCB81**	92	126
**PCB99**	90	176
**PCB101**	91	126
**PCB105**	102	138
**PCB110**	92	10
**PCB114**	97	130
**PCB126**	97	139
**PCB138**	98	114
**PCB146**	94	127
**PCB151**	84	119
**PCB156**	100	141
**PCB157**	94	131
**PCB167**	98	137
**PCB169**	99	142
**PCB (170 + 190)**	97	137
**PCB177**	97	134
**PCB180**	97	136
**PCB183**	94	131
**PCB187**	97	135

**Table 3:** Concentrations (μg/kg) of PCBs in PHA samples from municipal organic waste/sludge.

		Extraction Method		
	Raw PHA-Rich Biomass	NaClO	CHCl_3_	Biotrend
**PCB28**	nd	nd	nd	nd
**PCB77**	nd	2.3 ± 0.0050	1.8 ± 0.0039	1.5 ± 0.046
**PCB81**	1.7 ± 0.029	1.6 ± 0.030	2.6 ± 0.031	nd
**PCB99**	47.3 ± 3.40	51 ± 2.6	48 ± 6.2	nd
**PCB101**	2.6 ± 0.82	4.9 ± 2.3	2.4 ± 1.3	3.4 ± 0.020
**PCB105**	1 ± 0.1	1.2 ± 0.040	1.3 ± 0.20	1.7 ± 0.029
**PCB110**	nd	nd	nd	nd
**PCB114**	1 ± 0.07	1 ± 0.06	1 ± 0.08	1.3 ± 0.013
**PCB126**	nd	nd	nd	1.7 ± 0.012
**PCB138**	2.7 ± 1.1	2.2 ± 0.48	2.9 ± 0.89	2.1 ± 0.89
**PCB146**	1.3 ± 0.010	1.4 ± 0.050	1.4 ± 0.051	1.6 ± 0.031
**PCB151**	0.90 ± 0.014	nd	nd	1.4 ± 0.010
**PCB156**	nd	nd	nd	2 ± 0.02
**PCB157**	nd	nd	nd	1.7 ± 0.011
**PCB167**	nd	nd	nd	1.7 ± 0.0039
**PCB169**	3.4 ± 0.70	1.7 ± 0.20	1.6 ± 0.13	1.9 ± 0.30
**PCB** **(170 + 190)**	nd	6.6 ± 0.020	nd	7.1 ± 0.010
**PCB177**	1.4 ± 0.079	1.5 ± 0.10	1.5 ± 0.10	1.6 ± 0.69
**PCB180**	1.8 ± 0.29	1.6 ± 0.20	1.7 ± 0.14	1.8 ± 0.0040
**PCB183**	1.4 ± 0.011	1.5 ± 0.050	1.5 ± 0.047	1.9 ± 0.012
**PCB187**	1.6 ± 0.25	1.4 ± 0.018	1.5 ± 0.050	1.5 ± 0.019

nd: not detectable.

**Table 4:** Concentrations (μg/kg) of PCBs in commercial PHA and in PHA samples from fruit waste extracted according to the Biotrend method.

	Fruit Waste	Commercial
**PCB28**	nd	nd
**PCB77**	nd	2 ± 0.005
**PCB81**	nd	2.3 ± 0.040
**PCB99**	nd	50 ± 2.6
**PCB101**	nd	2.7 ± 0.031
**PCB105**	nd	1.2 ± 0.020
**PCB110**	nd	nd
**PCB114**	nd	1.4 ± 0.019
**PCB126**	nd	1.5 ± 0.30
**PCB138**	1.4 ± 0.21	2.1 ± 0.35
**PCB146**	nd	1.5 ± 0.019
**PCB151**	nd	1.1 ± 0.10
**PCB156**	nd	1.9 ± 0.011
**PCB157**	nd	1.7 ± 0.0048
**PCB167**	nd	1.8 ± 0.029
**PCB169**	2.3 ± 0.45	1.7 ± 0.17
**PCB** **(170 + 190)**	8.5 ± 0.012	nd
**PCB177**	nd	1.7 ± 0.010
**PCB180**	nd	1.5 ± 0.10
**PCB183**	nd	1.8 ± 0.0057
**PCB187**	nd	1.4 ± 0.050

nd: not detectable.
